# Galangin Abrogates Ovalbumin-Induced Airway Inflammation via Negative Regulation of NF-**κ**B

**DOI:** 10.1155/2013/767689

**Published:** 2013-05-25

**Authors:** Wang-Jian Zha, Yan Qian, Yi Shen, Qiang Du, Fei-Fei Chen, Zhen-Zhen Wu, Xiao Li, Mao Huang

**Affiliations:** ^1^Department of Respiratory Medicine, The First Affiliated Hospital, Nanjing Medical University, 300 Guangzhou Road, Nanjing 210029, China; ^2^Department of Respiratory Medicine, The Second Affiliated Hospital, Nanjing Medical University, 121 Jiangjiayuan Road, Nanjing 210011, China; ^3^Department of Pathology, The First Affiliated Hospital, Nanjing Medical University, 300 Guangzhou Road, Nanjing 210029, China

## Abstract

Persistent activation of nuclear factor *κ*B (NF-*κ*B) has been associated with the development of asthma. Galangin, the active pharmacological ingredient from *Alpinia galanga,* is reported to have a variety of anti-inflammatory properties in vitro via negative regulation of NF-*κ*B. This study aimed to investigate whether galangin can abrogate ovalbumin- (OVA-) induced airway inflammation by negative regulation of NF-*κ*B. BALB/c mice sensitized and challenged with OVA developed airway hyperresponsiveness (AHR) and inflammation. Galangin dose dependently inhibited OVA-induced increases in total cell counts, eosinophil counts, and interleukin-(IL-) 4, IL-5, and IL-13 levels in bronchoalveolar lavage fluid, and reduced serum level of OVA-specific IgE. Galangin also attenuated AHR, reduced eosinophil infiltration and goblet cell hyperplasia, and reduced expression of inducible nitric oxide synthase and vascular cell adhesion protein-1 (VCAM-1) levels in lung tissue. Additionally, galangin blocked inhibitor of *κ*B degradation, phosphorylation of the p65 subunit of NF-*κ*B, and p65 nuclear translocation from lung tissues of OVA-sensitized mice. Similarly, in normal human airway smooth muscle cells, galangin blocked tumor necrosis factor-*α* induced p65 nuclear translocation and expression of monocyte chemoattractant protein-1, eotaxin, CXCL10, and VCAM-1. These results suggest that galangin can attenuate ovalbumin-induced airway inflammation by inhibiting the NF-*κ*B pathway.

## 1. Introduction

Allergic asthma prevalence has increased considerably in recent decades such that it is now one of the most common chronic disorders in the world [1], and thus the accompanying health care costs are major health and socioeconomic problems. Allergic asthma is a chronic, complex, inflammatory disease caused by inappropriate responses to inhaled allergens and is characterized by reversible obstruction of airway hyperresponsiveness (AHR), infiltration of inflammatory cells into lung tissues, mucus overproduction, allergen-specific IgE, and the overexpression of inflammatory cytokines, chemokines, and adhesion molecules [2]. Interleukin-(IL-)4 and IL-13 regulate the expression of IgE from B lymphocytes, whereas IL-5 plays a key role in eosinophilic inflammation [3]. Chemokines such as eotaxin (CCL11) are crucial to the delivery of eosinophils to the airways [4], and monocyte chemotactic protein 1 (MCP-1 or CCL2) has monocyte and lymphocyte chemotactic activity and stimulates histamine release from basophils [5]. CXCL10 stimulates mast cell infiltration into airway smooth muscle bundles and subsequently activated cytokine secretion and airway smooth muscle cell (ASMC) proliferation [6]. Vascular cell adhesion molecule 1 (VCAM-1) is one of the most important adhesion molecules involved in adhesions between both circulating and resident leukocytes and the ASMC [7]. Despite extensive research into this disease, the exact pathophysiological mechanisms remain unclear and current treatments are not satisfactory.

NF-*κ*B is induced by many factors involved in asthmatic inflammation, including allergen challenge, cytokines, chemokines, and bacterial and viral infection. NF-*κ*B induces the expression of many mediators, growth factors, receptors, and enzymes important in the inflammatory cascade, leading to a feed-forward enhancement of inflammation. Genetic studies also implicate the NF-*κ*B pathway in corticosteroid-resistant asthma [8]. Increased activation of NF-*κ*B has been demonstrated in the lungs after allergen challenge and in ASMC, epithelial cells, and macrophages of asthmatic patients [5, 9]. Choi et al. also reported that pretreatment of NF-*κ*B p65 antisense oligonucleotides results in a significant inhibition of established asthmatic reaction in a murine model [10]. The development of a new strategy to inhibit lung-specific NF-*κ*B activity would be a welcomed addition in the management of asthma.

Galangin (GA, 3,5,7-trihydroxyflavone), a member of the flavonol class of flavonoids, is the active constituent of the rhizome of *Alpinia galanga*, a plant closely related to the ginger family (Zingiberaceae). The rhizome is commonly used as traditional medicine for cough and for a variety of ailments like diabetes, cold, stomachache, and diarrhea in China. Previous studies indicate that galangin has multifaceted pharmacological activities that reduce the contractility of rat aortic smooth muscle [11] and pig bladder smooth muscle [12], inhibit inflammation in arthritis and macrophages by negative regulation of NF-*κ*B [13], and restrain tumor growth [14]. Given that asthma is characterized by airway inflammation and hyperreaction and that galangin has antiinflammatory and anti-contractile activities, we investigated the effects of galangin on allergic airway inflammation and AHR in BALB/c mice. Our results clearly indicate that galangin attenuates allergic airway inflammation by potentially inhibiting NF-*κ*B pathway.

## 2. Materials and Methods

### 2.1. Animals

Female BALB/c mice, aged 6 to 8 weeks and weighing 18–22 g each, were purchased from Vital River Laboratories (Beijing, China). The mice were housed in the animal facility of Nanjing Medical University that was maintained at 22–24°C with a 12-hour dark/light cycle. Mice were fed a commercial pelleted mouse food and given water *ad libitum* under specific pathogen-free conditions according to standard guidelines for the care and use of animals. All experiments involving animals and tissue samples were performed in accordance with the guidelines of the National Institutes of Health and Nanjing Medical University with all procedures (2008-0007) approved by the Institutional Animal Care and Use Committee of Nanjing Medical University (Nanjing, China).

### 2.2. Culture and Galangin Treatment of Normal Human ASMC

Normal human ASMC was purchased from ScienCell Research Laboratories (Carlsbad, CA, USA) and were cultured in Dulbecco's modified Eagle's medium (Invitrogen-Gibco, Paisley, Scotland) supplemented with 4 mM L-glutamine, 20 U/L penicillin, 20 *μ*g/mL streptomycin and 10% fetal bovine serum (HyClone, Logan, UT, USA). Presence of ASMC was confirmed by identifying the characteristic hill and valley morphology using light microscopy. Cells between passages 4 and 8 were used for experiments. After starvation, cells were cultured with 10 ng/mL tumor necrosis factor alpha (TNF-*α*, R&D Systems, Minneapolis, MN, USA), in the presence and absence of 10 *μ*M galangin (Sigma-Aldrich, St. Louis, MO, USA) for 0, 1, 10, and 30 minutes before nuclear protein was extracted for subsequent immunoblotting analysis. Total RNA was extracted after cells were cultured with 10 ng/mL TNF-*α*, or TNF-*α* and galangin (1 *μ*M and 10 *μ*M), or TNF-*α* and 10 *μ*M thiophene-3-carboxamide 1 (TPCA-1, Sigma-Aldrich), inhibitor of I*κ*B kinase *β*, for 8 hours. Nuclear protein fractions were extracted after cells were cultured with 10 ng/mL TNF-*α*, TNF-*α* and galangin (1 *μ*M and 10 *μ*M), and TNF-*α* and TPCA-1 (10 *μ*M) for 10 min.

### 2.3. Ovalbumin Sensitization/Challenge Protocol

**Figure 1:**
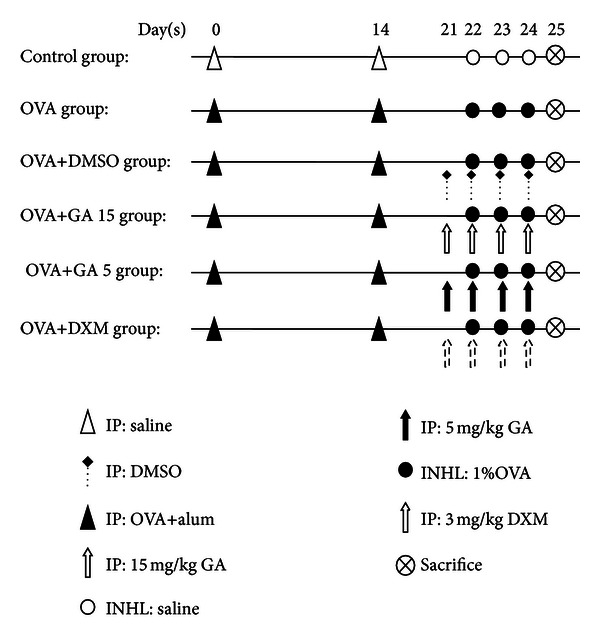
Experimental protocol. BALB/c mice were sensitized with 20 *μ*g OVA and 2 mg aluminum hydroxide gel in a total volume of 200 *μ*L by intraperitoneal injection on days 0 and 14. Mice were challenged with an aerosol of 1% in saline from day 22 to day 24. Control mice were sensitized and challenged using the same protocol but using only saline. Galangin, vehicle (DMSO), or DXM were given by intraperitoneal injection from day 21 to day 24. IP, intraperitoneal injection; INHL, inhalation.

Specific-pathogen-free female BALB/c mice were randomly divided equally into 6 groups: control, OVA, OVA+dimethyl sulfoxide (DMSO), OVA+GA 15 (galangin 15 mg/kg), OVA+GA 5 (galangin 5 mg/kg), and OVA+dexamethasone (DXM, 3 mg/kg). The mice were sensitized on days 0 and 14 by intraperitoneal injection of 20 *μ*g OVA (Grade V, Sigma-Aldrich) emulsified in 2 mg aluminum hydroxide gel (InvivoGen, San Diego, CA, USA) in a total volume of 200 *μ*L. On days 22, 23, and 24 after initial sensitization, the mice were challenged for 30 min with an aerosol of 1% (wt/vol) OVA in 0.9% saline using an ultrasonic nebulizer (NE-U11B; Omron Corp., Tokyo, Japan). Control mice were sensitized and challenged using the same protocol but using only saline. Galangin (15 and 5 mg/kg; Sigma-Aldrich) or vehicle (20 *μ*L DMSO in a total of 200 *μ*L saline) was given by intraperitoneal injection from day 21 to day 24. For a drug control, 3 mg/kg DXM (Sigma-Aldrich) was administered in the same manner. The procedures for allergen sensitization and treatment are summarized in Figure 1.

### 2.4. Measurement of Airway Responsiveness

Airway responsiveness to acetylcholine chloride (Ach) was evaluated by using an AniRes animal lung function analysis system (Synol High-Tech, Beijing, China) as previously described [15]. Briefly, the mice were anaesthetized by intraperitoneal injection of pentobarbital sodium (70 mg/kg) 24 h after the final challenge. A plastic tube (2 mm internal diameter) was inserted into the trachea via tracheotomy and a 27-gauge needle was inserted into the caudalis vein for drug administration. The mice were placed in a whole body plethysmography chamber and ventilated mechanically at a rate of 90 breaths/min with a tidal volume of 6 mL/kg. After establishment of stable airway pressure recording, ACh was administered intravenously with a microinfusion pump at a rate of 36 mL/h in progressively increasing doses (10, 30, 90, and 270 *μ*g/kg). After the administration of each dose, data were continuously collected from 5 sec to 1 min and maximum values of lung resistance (LR) were taken to express the changes in the airway function of the mice.

### 2.5. Analysis of Bronchoalveolar Lavage Fluid (BALF) and Serum

After measurement of AHR, the mice were bled by retroorbital puncture using sterile capillary tubes. Blood was allowed to clot for 30 minutes at room temperature before centrifugation for 15 min at 1000 ×g. Aliquots of serum were stored at −70°C until analysis. Immediately after blood collection, thoracic cavities were carefully opened. Tracheas were exposed, and BALF was collected by cannulating the upper part of the trachea and lavaging with ice-cold phosphate-buffered saline (PBS; 0.4 mL × 3; 85%–90% of the lavage volume was recovered). Lavaged samples from each mouse were centrifuged at 1000 ×g for 5 min at 4°C. Total number of inflammatory cells in BALF was counted with a hemocytometer. Smears of BALF cells were stained with Wright's staining for the differential cell count. The cells in the BALF were counted by two independent investigators in a single-blind study, which analyzed at least 200 cells each from four different random locations using a microscope. The levels of IL-4 (R&D Systems), IL-5, and IL-13 (ExCell Biology, Shanghai, China) in BALF and OVA-specific serum IgE (Shibayagi, Gunma, Japan) were determined by enzyme-linked immunosorbent assay according to the manufacturer's instructions. 

### 2.6. Lung Histology

For the histological evaluation of lung tissue, the right lungs obtained from sacrificed mice were immersed in 4% paraformaldehyde prior to embedding in paraffin. A series of microsections (5 *μ*m) were cut on a microtome and stained with hematoxylin and eosin (H&E) to assess the inflammatory cell infiltration, and periodic acid-Schiff (PAS) to quantify airway global cells. To determine the extent of mucus production, the mucus score was quantified using a 5-point grading system by a blinded scorer: 0, no goblet cells; 1, <25%; 2, 25%–50%; 3, 50%–75%; 4, >75%. Mucus score was performed in at least three different fields for each lung section [16]. 

Immunostaining for inducible nitric oxide synthase (iNOS) and VCAM-1 was performed using ultrasensitive immunohistochemistry S-P kits (Maixin Biology Corporation, Fuzhou, China). Sections (4 mm thick) were dewaxed, washed in Tris-buffered saline (TBS), and incubated with 3%  H_2_O_2_ in methanol for 20 min to block endogenous peroxidases. Sections were washed with TBS and incubated with 5% skim milk in TBS for 20 min. Blocked sections were incubated with anti-iNOS polyclonal antibody (Abcam, Cambridge, UK) or anti-VCAM-1 polyclonal antibody (Santa Cruz, CA, USA) for 90 min at 37°C. Secondary antibody was applied to each section for 10 min at room temperature, and the slides were rinsed in PBS (pH 7.4) three times after every incubation step. The slides were counterstained with hematoxylin, mounted, and observed under light microscopy.

### 2.7. Western Blot Analysis

Cytosolic or nuclear extractions from fresh lung tissues were obtained using NE-PER Nuclear and Cytoplasmic Extraction Reagents (Thermo Scientific, Rockford, IL, USA) and whole cell extractions were obtained using RIPA lysis buffer (Thermo Scientific) in the presence of protease inhibitors and phosphatase inhibitor (Roche, Indianapolis, IN, USA). Protein concentrations were determined using the BCA protein assay (Thermo Scientific). Denatured samples were loaded on a 10% SDS-PAGE gel. After electrophoresis, separated proteins were transferred to polyvinylidene difluoride membranes (Millipore, Billerica, MA, USA) by the wet transfer method. Nonspecific sites were blocked with 5% nonfat milk in TBS Tween 20 (TBST; 25 mM Tris pH 7.5, 150 mM NaCl, 0.1% Tween 20) for 2 h, and blots were incubated with anti-p65 antibody, antiphosphorylated p65 antibody, anti-I*κ*B*α* antibody, antiglyceraldehyde-3 phosphate dehydrogenase (GAPDH) antibody, anti-Histone H3 antibody (Cell Signaling Technology Inc., Beverly, MA, USA), anti-iNOS antibody (Abcam, Cambridge, UK), and anti-VCAM-1 antibody (Santa Cruz Biotechnology, Santa Cruz CA, USA) overnight at 4°C. Goat anti-rabbit horseradish peroxidase-conjugated IgG or rabbit anti-goat horseradish peroxidase-conjugated IgG was used to detect binding of the antibodies. After treating the membranes with enhanced chemiluminescence system reagents (GE Healthcare Bio-Sciences AB, Sweden), the binding of the specific antibody was visualized by the ChemiDoc MP system (Bio-Rad, Hercules, CA, USA).

### 2.8. Extraction of Total RNAS, Reverse Transcription-Polymerase Chain Reaction (RT-PCR), and Quantitative Real-Time PCR Analysis

Total lung RNA was extracted with TRIzol reagent (Invitrogen, Carlsbad, CA, USA) and reverse transcribed with PrimeScript RT Master Mix (Takara, Dalian, China). mRNA expression was qualified by means of quantitative real-time PCR (ABI PRISM 7500, USA) with SYBR Premix Ex Taq (Takara) using the recommended reaction conditions: 95°C for 10S, the 40 cycle of 95°C for 5S, and 60°C for 34S. Specific primers of human MCP-1, eotaxin, CXCL10, VCAM-1, and GAPDH were designed according to their published sequences using the Primer-BLAST online primer design software and synthesized by Invitrogen. Melting curve analysis was carried out to ensure the presence of one specific PCR product. Relative abundance of gene expression was normalized to GAPDH expression. The sequences of the gene specific primer sets were MCP-1, 5′CAGCCAGATGCAATCAATGCC3′ and 5′TGGAATCCTGAACCCACTTCT3′; eotaxin, 5′CCCCTTCAGCGACTAGAGAG3′ and 5′TCTTGGGGTCGGCACAGAT3′; CXCL10, 5′GTGGCATTCAAGGAGTACCTC3′ and 5′TGATGGCCTTCGATTCTGGATT3′; VCAM-1, 5′GGGAAGATGGTCGTGATCCTT3′ and 5′TCTGGGGTGGTCTCGATTTTA3′; GAPDH, 5′CCACTCCTCCACCTTTGAC3′ and 5′ACCCTGTTGCTGTAGCCA3′.

### 2.9. Statistical Analysis

Data are expressed as means ± standard error of the mean (SEM). Results were analyzed using one-way analysis of variance for repeated measures, followed by a Dunnett post hoc test to determine differences between treatment groups. Significant levels were set at *P* < 0.05.

## 3. Results

### 3.1. Galangin Reduces Airway Hyperreactivity in OVA-Sensitized Mice

**Figure 2:**
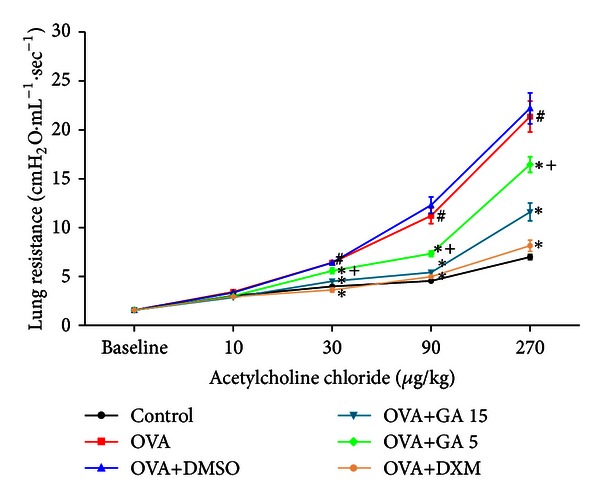
Treatment with galangin decreases the development of AHR in a murine model of asthma. Data are expressed as means ± SEM (*n* = 6 per group). ^#^ *P* < 0.05 compared with the control group; *P < 0.05 compared with the OVA group, ^+^ *P* < 0.05 compared with the OVA+GA 15 group.

To investigate the effect of galangin on AHR in response to increasing concentrations of Ach, we measured the LR in anaesthetized mice using invasive whole body plethysmography. There were no significant differences in baseline airway resistance among the six groups. The LR generated by the administration of Ach at doses from 30 to 270 *μ*g/kg increased dramatically in the OVA group compared with the control mice (Figure 2). The OVA group had significantly greater airway resistance than the control group. Treatment with galangin and DXM resulted in a sharp decrease in airway resistance compared with the OVA group, implying that in vivo inflammation mediated airway pathology was alleviated. However, the lower dose of galangin did not decrease lung resistance as striking as the higher concentration of galangin.

### 3.2. Galangin Attenuates Allergic Airway Inflammation in OVA-Sensitized Mice

**Figure 3:**
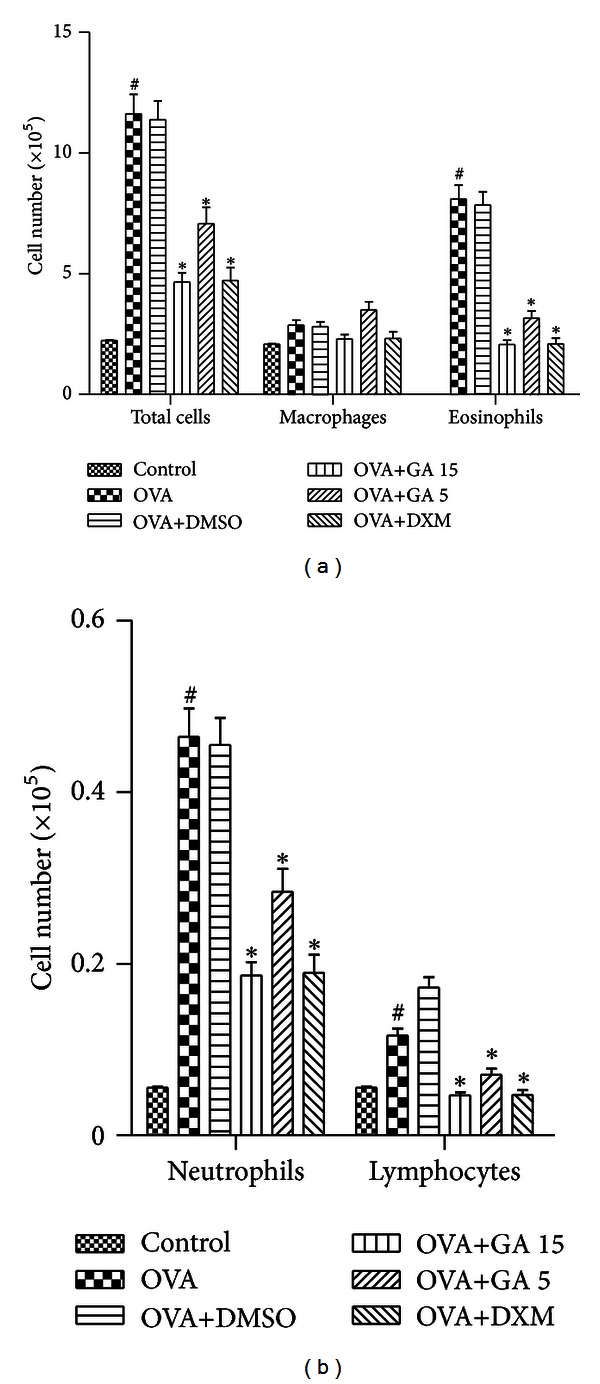
Treatment with galangin inhibits inflammatory cell accumulation in bronchoalveolar lavage fluid (BALF) in a murine model of asthma. Data are expressed as means ± SEM (*n* = 6 per group). ^#^ *P* < 0.05 compared with the control group; *P < 0.05 compared with the OVA group.

The total cell counts and differential cell counts in the BALF were evaluated 24 h after the last OVA challenge. In comparison with control group mice, the numbers of total leukocytes, eosinophils, neutrophils, and lymphocytes were markedly elevated in the OVA group. Treatment with galangin before OVA aerosol challenge dose dependently prevented this increase (Figure 3), and the vehicle (OVA+DMSO group) did not show any improvement. 

**Figure 4:**
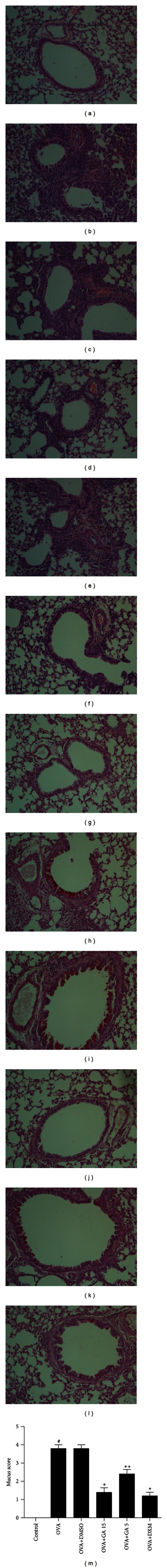
Treatment with galangin reduces inflammatory cells infiltration and goblet cell hyperplasia in lung tissue (magnification ×200). H&E staining of lung tissue from control group (a), OVA group (b), OVA+DMSO group (c), OVA+GA 15 group (d), OVA+GA 5 group (e), and OVA+DXM group (f). Lung sections were stained with PAS stain to analyze goblet cell hyperplasia from control group (g), OVA group (h), OVA+DMSO group (i), OVA+GA 15 group (j), OVA+GA 5 group (k), and OVA+DXM group (l). Graphs represent the mucus score (m) are expressed as means ± SEM (*n* = 6 per group). ^#^ *P* < 0.05 compared with the control group, *P < 0.05 compared with the OVA group, ^+^ *P* < 0.05 compared with the OVA+GA 15 group.

Lung sections stained by H&E were also evaluated for the development of airway inflammation. The OVA group exhibited an obvious peribronchial and perivascular inflammatory cell infiltration compared with the control group, and most infiltrating inflammatory cells were eosinophils (Figure 4). In asthmatic mice treated with DXM and higher dose of galangin (15 mg/kg), eosinophil infiltration was significantly alleviated compared with those in the OVA group, while no significant effects were shown in lower dose of galangin (5 mg/kg) treated mice (Figure 4(e)). On the other hand, OVA-challenged mice developed marked goblet cell hyperplasia and mucus hypersecretion in the lumen of the bronchioles. In contrast, OVA+GA group mice and OVA+DXM group mice showed a reduction in the number of PAS-stained goblet cells (Figure 4). However, the lower dose of galangin did not reduce goblet cells as dramatically as the higher concentration of galangin (Figure 4(m)). 

### 3.3. Galangin Reduces the Levels of Th2 Cytokine in BALF and OVA-Specific IgE in Serum

**Figure 5:**
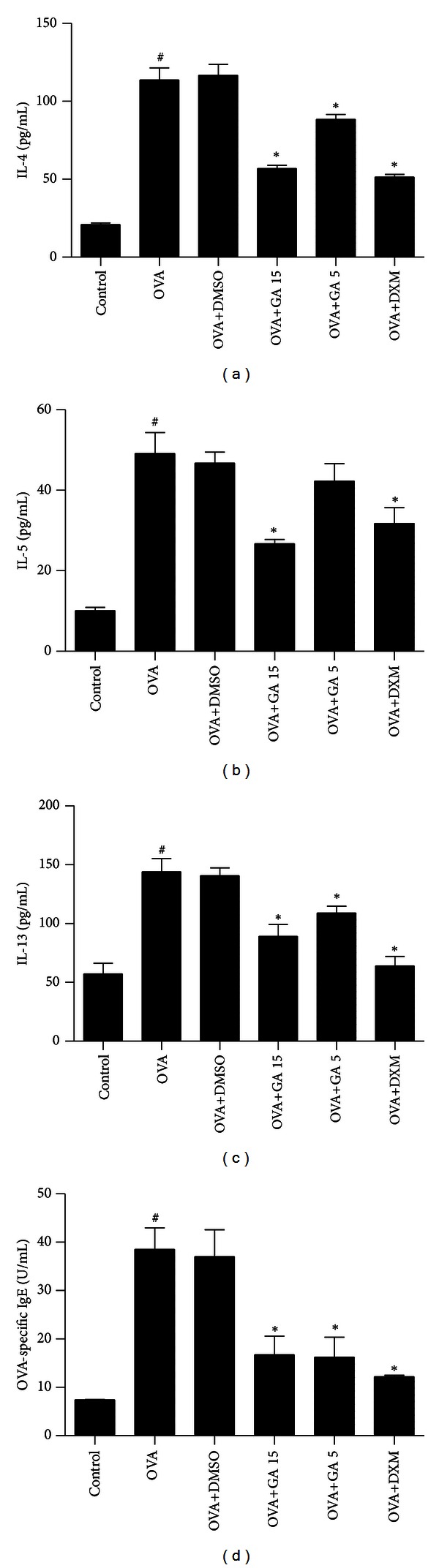
Treatment with galangin reduces levels of Th2 cytokine in BALF and OVA-specific IgE in serum in a murine model of asthma. Concentrations of IL-4 (a), IL-5 (b), IL-13 (c), and OVA-specific IgE (d) were measured by enzyme-linked immunosorbent assay. Data are expressed as means ± SEM (*n* = 6 per group). ^#^ *P* < 0.05 compared with the control group; *P < 0.05 compared with the OVA group.

IL-4, IL-5, and IL-13 in the BALF and OVA-specific IgE in serum were notably increased in the OVA group compared with the control group. Administration of galangin dose dependently reduced the levels of T-helper type 2 (Th2) cytokines in BALF and OVA-specific IgE in serum compared with those in OVA group mice (Figure 5). However, no significant reduction of IL-5 in BALF was shown in OVA+GA 5 group mice (Figure 5(b)). This finding implies the galangin can modify Th2-predominant immune activity in OVA-induced mouse asthma model.

### 3.4. Galangin Suppresses NF-*κ*B Activity in OVA-Sensitized Mice

**Figure 6:**
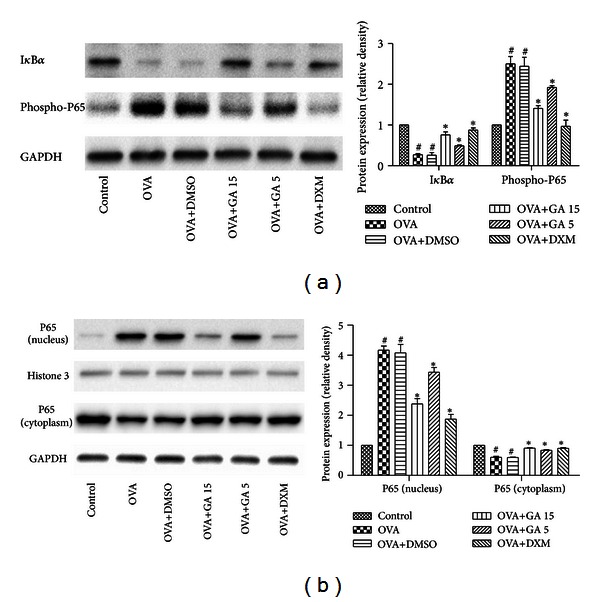
Treatment with galangin inhibits NF-*κ*B activity in OVA-sensitized mice. The level of I*κ*B*α* and phosphorylation of NF-*κ*B P65 in whole cells were measured by western blot (a). The level of NF-*κ*B P65 in nucleus and cytoplasm were measured by western blot (b). Data are expressed as means ± SEM (*n* = 6 per group). ^#^ *P* < 0.05 compared with the control group; *P < 0.05 compared with the OVA group.

To verify that the antiinflammatory effects of galangin in an OVA-induced mouse asthma model were mediated by the inhibition of NF-*κ*B, we examined degradation of I*κ*B*α*, phosphorylation of p65 subunit of NF-*κ*B, and nuclear translocation of p65 in lung tissues obtained 24 h after the final challenge. OVA challenge induced degradation of I*κ*B*α* and phosphorylation of p65 subunit, raised the level of p65 subunit in the nuclear extract of the lung tissue, and reduced the level of p65 in the cytoplasm extract. Galangin dose dependently reduced degradation of I*κ*B*α*, phosphorylation of p65, and nuclear translocation of p65 (Figure 6), suggesting that galangin may exert its anti-inflammation actions via suppression of NF-*κ*B activity.

### 3.5. Galangin Decreases iNOS Expression in OVA-Sensitized Mice

**Figure 7:**
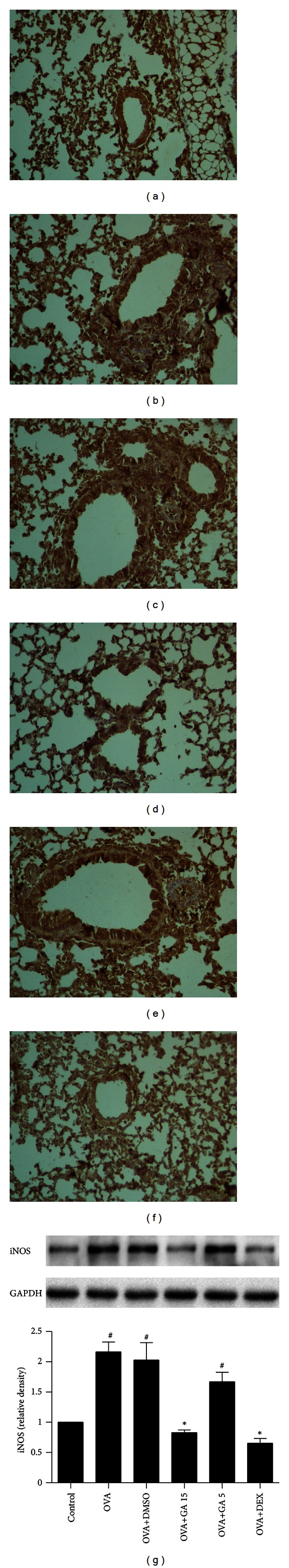
Treatment with galangin suppresses expression iNOS in OVA-sensitized mice. iNOS expression was analyzed by immunohistochemistry staining in control group (a), OVA group (b), OVA+DMSO group (c), OVA+GA 15 group (d), OVA+GA 5 group (e), and OVA+DXM group (f) (magnification ×200). iNOS protein levels were detected by western blot in lung tissue (g). Data are expressed as means ± SEM (*n* = 6 per group). ^#^ *P* < 0.05 compared with the control group; *P < 0.05 compared with the OVA group.

To determine whether iNOS was related to the antiinflammatory effects of galangin, we investigated iNOS expression in lung tissue. As shown in Figure 7, OVA sensitization increased iNOS expression in the lung compared with saline-challenged mice. Strong staining was mainly localized to bronchial epithelial cells, ASMC, and the infiltrating inflammatory cells around the bronchi. iNOS was dramatically decreased in OVA+GA 15 group and OVA+DXM group mice, but no such effect was observed in OVA+GA 5 group mice. Western blot analysis of whole lung tissue also revealed a significant reduction of iNOS expression in mice treated with higher dose of galangin and DXM. 

### 3.6. Galangin Decreases VCAM-1 Expression in OVA-Sensitized Mice

**Figure 8:**
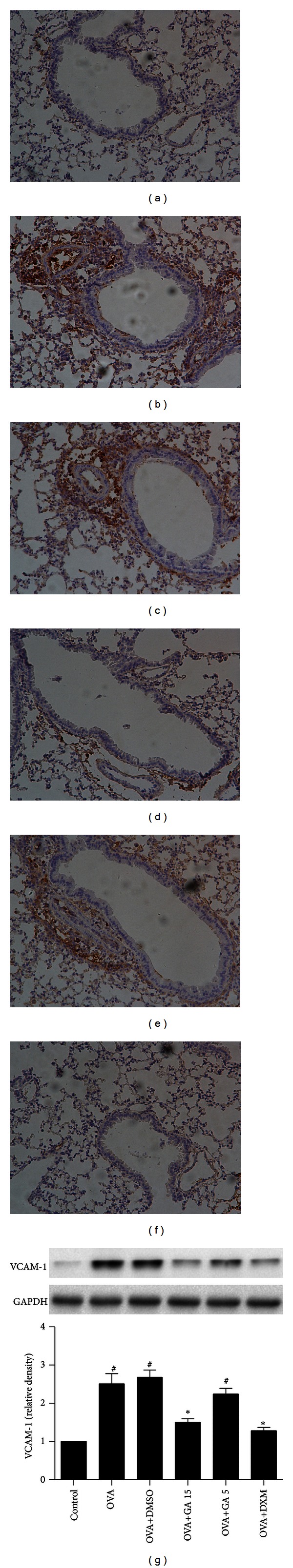
Treatment with galangin suppresses VCAM-1 expression in OVA-sensitized mice. VCAM-1 expression was analyzed by immunohistochemistry staining in control group (a), OVA group (b), OVA+DMSO group (c), OVA+GA 15 group (d), OVA+GA 5 group (e), and OVA+DXM group (f) (magnification ×200). VCAM-1 protein levels were detected by western blot in lung tissue (g). Data are expressed as means ± SEM (*n* = 6 per group). ^#^ *P* < 0.05 compared with the control group; *P < 0.05 compared with the OVA group.

To further explore the possible protective mechanism underlying the activity of galangin in airway inflammation, we investigated the expression of VCAM-1, one of the adhesion molecules related to infiltration of inflammatory cells in lungs of OVA-challenged mice. As shown in Figure 8, OVA-sensitized mice showed a significant increase in expression of VCAM-1 in airway smooth muscle cells, alveolar epithelial cells, and vascular endothelial cells compared with control group mice. However, in OVA+GA 15 group and OVA+DXM group mice, we observed a substantial reduction in the expression of VCAM-1 compared with OVA-challenged mice, while no such effect was observed in OVA+GA 5 group mice. Western blot analysis of whole lung tissue also revealed a significant reduction of VCAM-1 expression in mice treated with higher dose of galangin and DXM.

### 3.7. Galangin Inhibits TNF-*α*-Induced NF-*κ*B Activation in Normal Human ASMC

**Figure 9:**
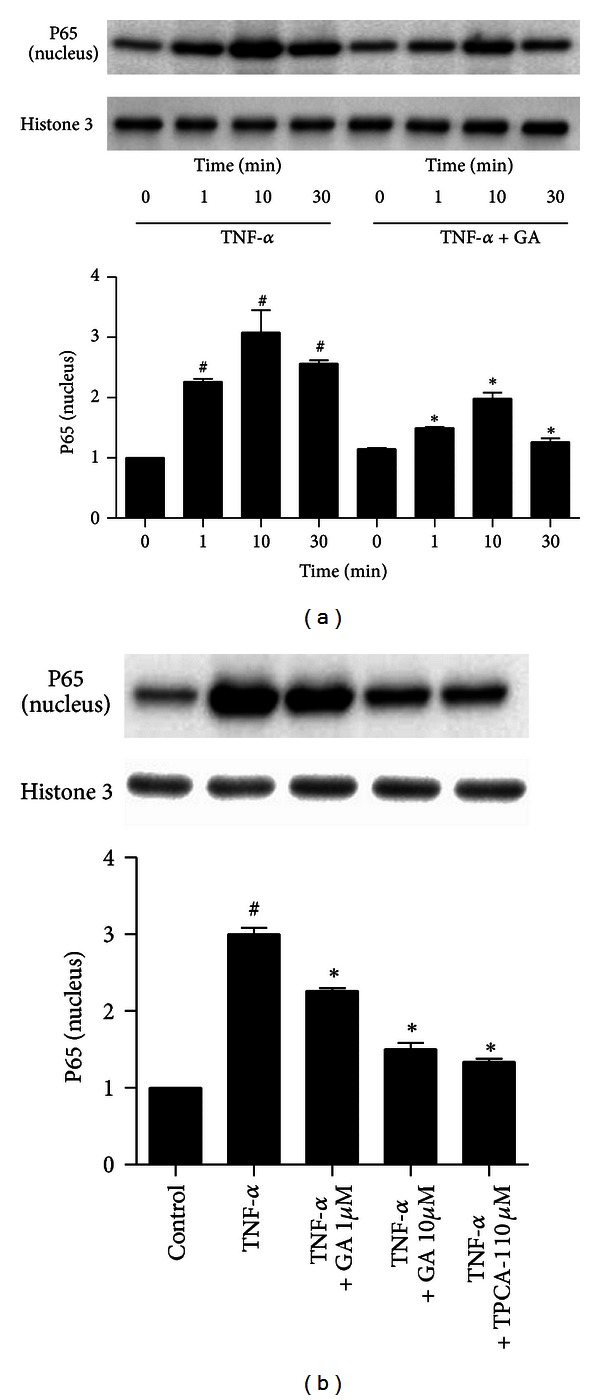
Treatment with galangin suppresses TNF-*α*-induced NF-*κ*B activation in normal human ASMC. ASMC were cultured with 10 ng/mL TNF-*α* in the presence or absence of 10 *μ*M galangin for 0, 1, 10, and 30 minutes. The level of NF-*κ*B P65 in the nucleus was measured by western blot (a). Cells were cultured with 10 ng/mL TNF-*α*, or TNF-*α* and galangin (1 *μ*M and 10 *μ*M), or TNF-*α* and 10 *μ*M TPCA-1. The level of NF-*κ*B P65 in the nucleus was measured by western blot (b). Data are expressed as means ± SEM of three experiments. ^#^ *P* < 0.05 compared with the control group; *P < 0.05 compared with TNF-*α* alone.

**Figure 10:**
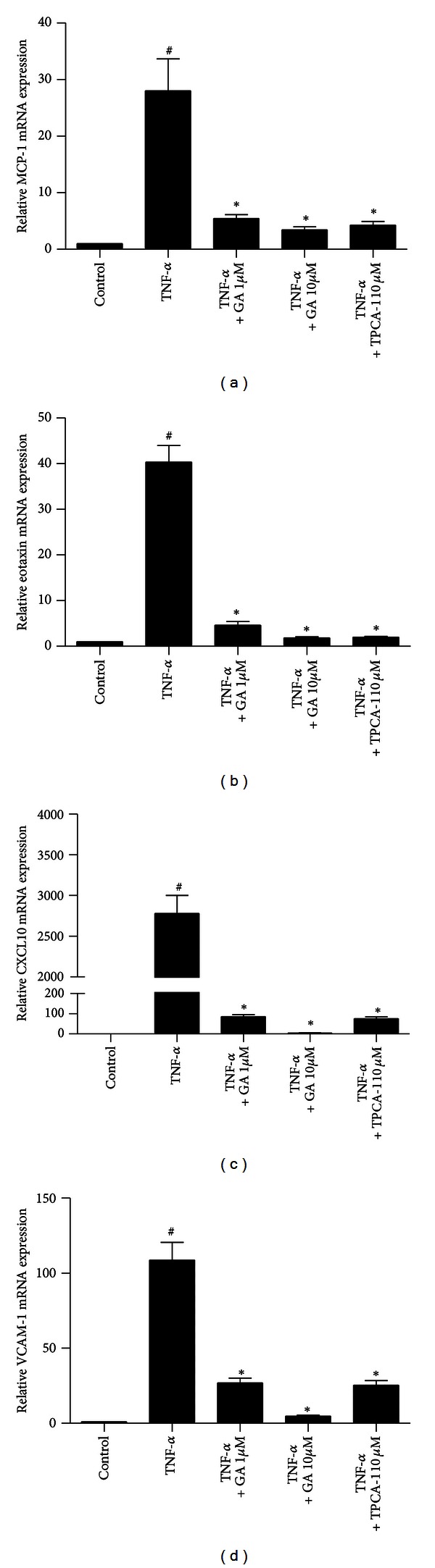
Treatment with galangin suppresses TNF-*α*-induced upregulation of MCP-1, eotaxin, CXCL10, and VCAM-1 mRNA expression in normal human ASMC. Cells were cultured with 10 ng/mL TNF-*α*, or TNF-*α* and galangin (1 *μ*M and 10 *μ*M), or TNF-*α* and 10 *μ*M TPCA-1 before total RNA was extracted. Real-time PCR was used to measure expression of MCP-1 (a), eotaxin (b), CXCL10 (c), and VCAM-1 (d). Data are expressed as means ± SEM of three experiments. ^#^ *P* < 0.05 compared with the control group; *P < 0.05 compared with TNF-*α* alone.

ASMC released chemokines and adhesion molecules to induce an inflammatory response and eosinophil migration in asthma patients [5, 6, 17, 18]. To further ascertain the antiinflammatory mechanism of galangin in relevant airway cell types, we studied the effects of galangin on TNF-*α*-induced activation of NF-*κ*B, upregulation of MCP-1, eotaxin, CXCL10, and VCAM-1 in ASMC. TNF-*α* induced a rapid nuclear translocation of p65 within 1 min, the level of p65 in the nucleus reached the maximum concentration at the time of 10 min, and p65 began to migrate to the cytoplasm. Galangin can dose dependently decrease p65 nuclear translocation (Figure 9). Furthermore, galangin can block TNF-*α*-induced upregulation of MCP-1, eotaxin, CXCL10, and VCAM-1 mRNA expression in normal human ASMC, as well as inhibitor of I*κ*B kinase *β*, TPCA-1 (Figure 10).

## 4. Discussion

NF-*κ*B regulates host inflammatory and immune responses by increasing the expression of specific cellular genes. These include genes encoding at least 27 different cytokines and chemokines, receptors involved in immune recognition such as members of the major histocompatibility complex, proteins involved in antigen presentation, and receptors required for inflammatory cells adhesion and migration [19]. Cytokines that are stimulated by NF-*κ*B, such as IL-1*β* and TNF-*α*, can also directly activate the NF-*κ*B pathway, thus establishing a positive autoregulatory loop that can amplify the inflammatory response and increase the duration of chronic inflammation. NF-*κ*B also stimulates the expression of enzymes, the products of which contribute to the pathogenesis of the inflammatory process, including iNOS. Persistent NF-*κ*B activation has been observed in allergic airway inflammation in both human and animal models of asthma [16, 20]. In addition, TNF-*α*-stimulation of ASMC triggers NF-kB–dependent gene expression [5, 6, 17, 18]. Stimulation of the NF-*κ*B pathway is mediated via diverse signal transduction cascades. These signals activate the I*κ*B kinases, IKK*α* and IKK*β*, which phosphorylates inhibitory proteins known as I*κ*B*α* to result in their ubiquitination and degradation by the proteasome. The degradation of I*κ*B*α* results in the translocation of NF-*κ*B from the cytoplasm to the nucleus where it activates the expression of specific cellular genes [21]. Various therapy strategies targeted at the NF-*κ*B signaling pathway such as p65-specific antisense and small interfering ribonucleic acid [10, 22], NF-*κ*B-specific decoy oligonucleotide [23], IKK*β*-selective small molecule inhibitors [24], and proteasome inhibitors [25] have demonstrated beneficial effects in experimental asthma models. In the present study, we found that galangin inhibited I*κ*B*α* degradation and p65 phosphorylation and nuclear translocation in an OVA-induced mouse asthma model. ASMC contribute to the pathogenesis of asthma at multiple levels beyond its contractile functions; they secrete cytokines, chemokines, and adhesion molecules, which may participate in or even perpetuate mucosal inflammatory changes via the activation and recruitment of inflammatory cells [26]. In an in vitro study, we founded that galangin reduced the expression of chemokines and adhesion molecules thorough inhibition of the NF-*κ*B pathway in TNF-*α*-stimulated normal human ASMC. In conclusion, herein we made the novel observation that galangin, the main compound of galangal, can dosedependently suppress various aspects of OVA-induced Th2-mediated allergic airway inflammation in mice via inhibition of NF-*κ*B activity.

Th2 cells play an essential role in the initiation and progression of allergic asthma by induction of cytokine secretion, and NF-*κ*B is more than a critical transcription factor for Th2 cell differentiation [27], and it also plays a seminal role in asthma by regulating the transcriptional process of NF-*κ*B-dependent gene encoding protein such as IL-4, IL-5, IL-13, eotaxin, CXCL10, MCP-1, and VCAM-1 [28]. In particular, Th2 cytokines IL-4, IL-5, and IL-13 lead to eosinophil-rich inflammation in lungs, enhanced IgE production, and mucus hypersecretion by epithelial goblet cells [29]. However, galangin treatment significantly reduced levels of IL-4, IL-5, and IL-13 in BALF and OVA-specific IgE in serum of OVA sensitized mice. In addition, galangin prevented the elevation of inflammatory cells such as eosinophils, neutrophils, and lymphocytes. These results were confirmed by histological analysis, which showed that galangin inhibited inflammatory cell infiltration and mucus hypersecretion. Similar findings were noted in OVA-challenged mice with therapy targeted at the NF-*κ*B signaling pathway [10, 22–25]. The above data indicate that the antiinflammatory effects of galangin may be due to suppression of NF-*κ*B activation in the inflammatory cells.

Increased NO levels in the airways of both rodents and human asthma patients may be an ideal indicator of asthma severity [30]. Measurement of exhaled NO (eNO) has been suggested as helpful for monitoring airway inflammation in asthma, especially in cases of exacerbated asthma [31]. eNO levels directly depend on NOS to generate NO. There are several species of NOS including constitutive NOS, iNOS, and endothelial NOS. iNOS is believed to be the primary enzyme responsible for generation of eNO in inflamed airways [32]. IL-13, a Th2 cytokine, robustly induces the expression of an active dimeric iNOS enzyme in primary human bronchial epithelial cells consistent with its expression in relation to Th2 inflammation [33]. Determining the source of NO in asthma may be of help in the elucidation of its pathogenic role and provide an alternative therapy. The dual effects of NO in many pathological conditions are likely related to its source, concentration, and site of action. In this process, excessive NO may increase eosinophils in the airway and shift the immune balance toward Th2 cells, thus exacerbating inflammation in the airway. Inhibiting iNOS can reduce resistance of the respiratory system response, eosinophilic and mononuclear cell recruitment, and collagen and elastic fibers content in airways [34]. Additionally, iNOS gene expression is regulated by the NF-*κ*B pathway [35]. Our results show that galangin markedly suppressed OVA-induced iNOS expression in the lungs, which may be due to the direct inhibition of the NF-*κ*B pathway and the reduced levels of IL-13 in the allergic airways.

Upregulation of VCAM-1 involves adhesions between circulating and resident leukocytes and human ASMC during airway inflammatory reaction [7]. According to previous studies, VCAM-1 is up-regulated after bronchial allergen challenge in patients with asthma [36]. Additionally, it was reported that cytokines such as IL-4 activate the rapid expression of VCAM-1 [37]. In this study, we found that the expression of VCAM-1 in lung tissues and IL-4 in BALF was significantly increased after OVA challenge. However, mice treated with galangin showed decreased expression of VCAM-1 accompanied by a reduction of IL-4 compared with OVA-challenged mice. In addition, VCAM-1 gene expression is regulated by the NF-*κ*B pathway [28]. These findings demonstrate that administration of galangin attenuates inflammatory cells infiltration, via partial downregulation of VCAM-1 through the NF-*κ*B pathway.

AHR is a fundamental abnormality in asthma. There are many potential factors contributing to the excessive airway response demonstrable on airway challenge, one of the most important being chronic airway inflammation. eNO, which is primarily derived from iNOS in inflamed airways, has been shown to correlate with sputum eosinophils and eosinophils in airway biopsies, and a strong correlation has been shown between eNO and AHR to methacholine [38]. Th2 cytokines, particularly IL-4, which regulate inflammatory cell recruitment to the lung, are critical in allergic inflammation and the development of AHR [39]. In addition, eosinophils are crucial in the development of AHR, because an association between peripheral blood eosinophil activation and AHR has also been demonstrated [40]. AHR is absent in mice whose eosinophils are completely ablated [41]. Calcium plays another key role in the initiation and maintenance of airway smooth muscle contraction and is closely coupled to AHR [42], given that it activates myosin light chain kinase in particular, as well as other kinases which participate in excitation-contraction coupling, such as RhoA kinase [43]. Galangin also has multifaceted pharmacological activities to reduce the contractility of rat aortic smooth muscle [11] and pig bladder smooth muscle [12] through the inhibition of calcium influx and the modulation of intracellular calcium movement. Our findings demonstrated, for the first time, that treatment with galangin significantly inhibited AHR in a model of allergic asthma. Inhibition of AHR may be attributed to reduction of iNOS, Th2 cytokines, and eosinophilic airway inflammation in allergic mice. Further investigation into the calcium channels pathway is needed.

## 5. Conclusions

We demonstrated the potential therapeutic action of galangin in an experimental model of asthma and its antiinflammatory properties in human ASMC. The main findings are that (I) galangin reduced airway hyperreactivity, (II) suppressed both the infiltration of inflammatory cells and hyperplasia of goblet cells to the airways, (III) attenuated the elevated levels of IL-4, IL-5, and IL-13 in BALF and OVA-specific IgE in serum, (IV) suppressed the NF-*κ*B activity and iNOS, VCAM-1 expression in OVA sensitized mice, and (V) inhibited TNF-*α*-induced NF-*κ*B activation in normal ASMC. These findings support a therapeutic value for galangin in the treatment of asthma.
